# Supplementary material for: Urinary Exosomes of Patients with Cystic Fibrosis Unravel CFTR-Related Renal Disease
Source: Int J Mol Sci. 2020 Sep 10;21(18):6625. doi: 10.3390/ijms21186625 (PMC7554933; doi:10.3390/ijms21186625)
Supplement: Supplementary file 1 [file ijms-21-06625-s001.zip › ijms-904635 (supplementary figures).pdf]

Supplemental Figure 1

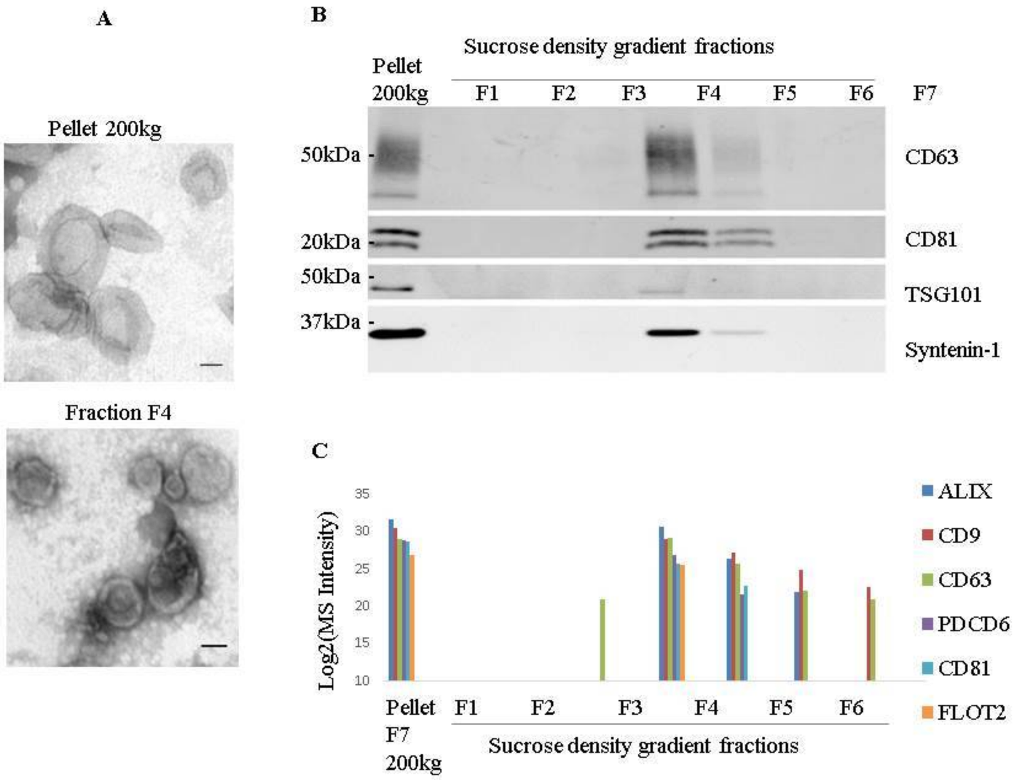

Figure S1. Urinary exosomes are enriched and purified by density gradient fractionation. **A**. Transmission electron microscopy images showing the typical "cup-shape" exosomal structures in the 200kg ultracentrifigation pellet (top image) and in the F4 fraction from sucrose density gradient (bottom image). Scale bar = 50 nm. **B**. Representative Western Blot experiment detecting specific exosomal proteins CD63, CD81, TSG101 and Syntenin-1 in pellet 200kg, and F4 and F5 fractions of sucrose density gradient. Following SDS-PAGE and transfer of proteins, the nitrocellulose membrane was cut into fragments to separate proteins of different molecular weight and specific exosomal markers were immuno-detected on appropriate membrane fragments. Raw data are readily available. **C**. Mass spectrometry identification of exosomal markers, Programmed cell death 6-interacting protein (ALIX), CD9, CD63, Programmed cell death protein 6 (PDCD6), CD81, and Flotillin 2 in the pellet and different fractions.

Supplementary Figure 2

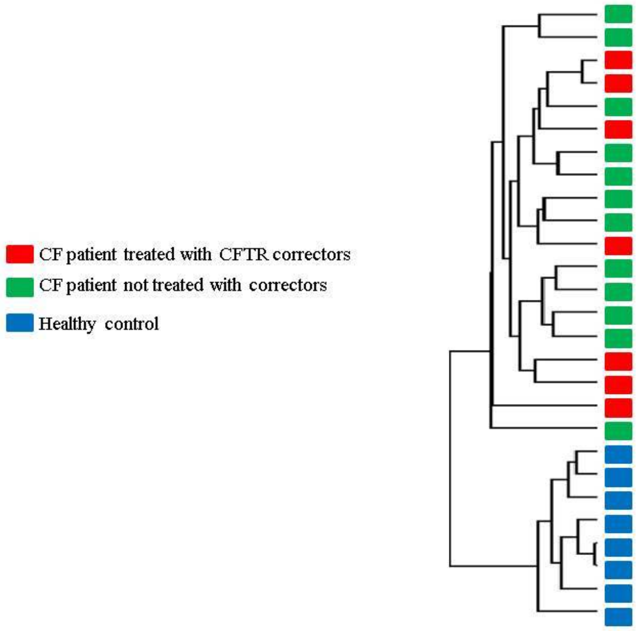

Figure S2. Dendogram of healthy control, patient (treated and untreated) according urinary exosomal proteomic content.
